# Relationship between Metal Exposures, Dietary Macronutrient Intake, and Blood Glucose Levels of Informal Electronic Waste Recyclers in Ghana

**DOI:** 10.3390/ijerph191912768

**Published:** 2022-10-06

**Authors:** Fayizatu Dawud, Sylvia Akpene Takyi, John Arko-Mensah, Niladri Basu, Godfred Egbi, Ebenezer Ofori-Attah, Serwaa Akoto Bawuah, Julius N. Fobil

**Affiliations:** 1 School of Public Health, University of Ghana, Legon, Accra P.O. Box LG13, Ghana; 2 McGill University, Montreal, QC H9X 3V9, Canada; 3 Noguchi Memorial Institute for Medical Research, University of Ghana, Legon, Accra P.O. Box LG 581, Ghana

**Keywords:** e-waste, metals, macronutrients, diabetes, Ghana

## Abstract

While metal exposures are generally high among informal electronic waste (e-waste) recyclers, the joint effect of metals and dietary macronutrients on their metabolic health is unknown. Therefore, we investigated the relationship between metal exposures, dietary macronutrients intake, and blood glucose levels of e-waste recyclers at Agbogbloshie using dietary information (48-h recall survey), blood metals (Pb & Cd), and HbA1C levels of 151 participants (100 e-waste recyclers and 51 controls from the Accra, Ghana) in March 2017. A linear regression model was used to estimate the joint relationship between metal exposures, dietary macronutrient intake, and blood glucose levels. Except for dietary proteins, both groups had macronutrient deficiencies. Diabetes prevalence was significantly higher among controls. Saturated fat, OMEGA-3, and cholesterol intake were associated with significant increases in blood glucose levels of recyclers. In a joint model, while 1 mg of cholesterol consumed was associated with a 0.7% increase in blood glucose, 1 g/L of Pb was found to significantly increase blood glucose levels by 0.9% among recyclers. Although the dietary consumption of cholesterol and fat was not high, it is still possible that exposure to Pb and Cd may still increase the risk of diabetes among both e-waste recyclers and the general population.

## 1. Introduction

Diabetes mellitus is a leading cause of death, blindness, and chronic kidney failure all over the world. Diabetes has also been linked to the incidence of several vascular diseases, including myocardial infarction, stroke, and peripheral vascular disorders [1,2,3]. In particular, diabetes mellitus develops owing to reduced insulin sensitivity, and insufficient insulin production. The insulin-dependent diabetes, which is often known as type 1 diabetes, is also a chronic disorder in which the pancreas produces little or no insulin on its own. Apart from genetic predisposition linked to diabetes, several research studies have also identified excessive calorie consumption, obesity, and lack of physical activity as key risk factors for the development of diabetes. For instance, the excessive intake of macronutrients (e.g., carbohydrates, fat, and protein) has been associated with the development of obesity and diabetes mellitus [4,5]. The consumption of fat-rich diets may alter insulin secretion and cause an impaired glucose balance [6,7,8].

Apart from dietary intake, the prevalence of diabetes appears to rise in tandem with exposure to several environmentally hazardous chemicals such as metals, which tend to increase with rapid industrial development. Globally, rapid technological progress coupled with rising demand for technology has resulted in massive volumes of electronic waste (e-waste) [9,10,11,12,13]. This type of waste contains hazardous chemicals, which pose significant harmful environmental impacts if not well disposed of or recycled. Particularly in Ghana, the informal techniques, such as manual dismantling, and open-air burning employed, unintentionally release metals (e.g., lead (Pb), cadmium (Cd), etc.), particulates (e.g., particulate matter (PM)), and organic compounds (e.g., dioxins, polyaromatic hydrocarbons (PAH)) into the environment. The US Environmental Protection Agency (EPA) has classified some of the metals associated with informal e-waste recycling as endocrine-disrupting chemicals (EDCs), which have the potential to alter metabolic processes in the body. Exposure to these environmental pollutants induces several adverse effects related to cardiovascular and respiratory systems when inhaled over time [14,15]. For instance, exposure to Pb may induce an increased release of reactive oxygen species (ROS), which, among many other effects, impair the normal function of the pancreatic cells and further enhances insulin resistance in the body [16,17]. Such impaired biological processes have been shown to influence blood glucose metabolism and regulation in humans [18].

A balanced diet, together with or in place of hypoglycemic medications, is an integral way of managing impaired metabolism of blood glucose [19,20]. The consumption of different diets with the varying nutritional content of macronutrients (including fats, carbohydrates, and proteins) may result in changes in metabolites and the gut microbiota, responsible for the entire body’s glucose metabolism [21]. Several amino acid containing-diets, such as those rich in high-protein foods (e.g., red meats), may alter plasma branched-chain-amino-acid (BCAA) concentrations, which are tied to an increased risk of type 2 diabetes [21,22]. Some diets have been reported to be associated with significant reductions in the effects of chemicals on metabolic diseases like hypertension [9]. For instance, iron-rich diets contain both antioxidant and anti-inflammatory properties, which may reduce the effects of oxidative stressors, associated with chemicals exposures, by terminating and inhibiting chain reactions of reactive oxygen species to regulate blood glucose [9,23]. However, to better understand the significance of dietary effects on blood glucose control, in-depth research into the effects of diet on glucose metabolism remains necessary.

Studies investigating the potential modifying effects of diets (macro and micronutrient) intake on environmental pollutant-induced diabetes among groups exposed to environmental pollutants are limited [22,24]. E-waste recyclers are therefore at a higher risk of developing metabolic disorders, such as diabetes, hypertension, and other non-communicable diseases, given their high risk of exposure to a myriad of deleterious chemicals. For instance, the exposure to Pb may induce oxidative stress and further promote insulin resistance, while Cd exposure may impair glucose homeostasis, thus increasing the risk of type 2 diabetes in humans [25,26]. The few studies that have examined these relationships, failed to consider informal e-waste recyclers, who are particularly at risk of exposure to a myriad of metals, given the nature of their job. In detail, informal e-waste recyclers are workers who employ low cost but dangerous recycling practices, such as open air burning, manual collection, dismantling, and sorting in managing waste electrical and electronic equipment. Although nutrition has been identified to play a mitigating role by reducing the effects of these pollutants on metabolic health, the macronutrient status of these e-waste recyclers is not known. Considering the rigorous and physically demanding nature of informal e-waste recycling activities, human exposure to these metals may serve as a risk for micronutrients depletion [27,28,29]. Thus, predisposing these recyclers to poor nutritional and blood glucose status. To better understand how diet (macro and micronutrients) can mitigate the negative effects of environmental contaminants on blood glucose, well-designed, rigorous research remains necessary. We therefore investigated the relationship between metal exposures, dietary macronutrients intake, and blood glucose levels of e-waste recyclers at Agbogbloshie. First, we measured metal (Pb and Cd) levels, dietary macronutrient intake as well as glycated hemoglobin (HbA1c) levels among waste recyclers at Agbogbloshie and a general population (Madina-Zongo) who are unexposed to e-waste recovery in order to better characterize how metal exposure due to informal e-waste recycling can influence blood glucose metabolism. We used the HbA1c levels as a prognostic measure for the prevalence of diabetes among the study population. Furthermore, the study investigated the joint association between metal exposures and dietary macronutrient intake among e-waste workers.

## 2. Materials and Methods

### 2.1. Data Source, Study Design and Site

In this study, we obtained and analyzed archived whole blood samples collected during GEOHealth II project (a longitudinal study with 3 reported rounds of data collection) for mean blood glucose levels (HbA1c) and metals, i.e., Lead (Pb) and Cadmium (Cd). For purposes of the study, we retrieved and analyzed blood samples from wave I, generated data on levels of glycated hemoglobin and concentration of metals in blood, using a cross-sectional design. We also obtained dietary information and nutritional status of the participants (e-waste recyclers and comparison group) to analyze and study the relationship between metal exposures (Pb and Cd), dietary macronutrient intake and blood glucose levels of informal e-waste recyclers at Agbogbloshie and a comparison group (Madina). The sample size was pre-determined by the parent project (GEOHealth II project). A total of 132 study participants were recruited during the GEOHealth II project (a longitudinal study with 3 reported rounds of data collection). Given the fore knowledge of a likely 10–20% attrition as indicated in literature for cohort studies, the broader project recruited; 100 recyclers (assigned to different job tasks at Agbogbloshie) and 50 controls from Madina-Zongo (~18 km from Agbogbloshie). At baseline [9,10,30], a total of 150 archived samples from participants of the GEOHealth II project was analyzed. Apart from whether or not participants partake in e-waste recycling, these two communities have similar demographic characteristics, such that residents are mostly from Ghana’s northern regions, are mostly Moslems, and eat similar types of foods.

Further, as detailed in our earlier publication, in line with the GEOHealth II project [9,10,13,28,30], participants were aged between 18 and above, who had worked at the e-waste site for at least six months. Participants from the control site (Madina-Zongo, Accra, Ghana) were similarly aged and had similar demographic characteristics as the e-waste recyclers (e.g., culture and dietary habits). Furthermore, participants from the control site must have lived there for a minimum of six months. The study protocols were approved by Institutional Review Boards at the University of Ghana, the University of Michigan, and McGill University [1,9,10,28,30].

### 2.2. Data Collection Techniques & Tools

#### 2.2.1. Anthropometric Measurements

Secondary data on weight, height, and calculated body mass index (BMI) were drawn from the GEOHealth II Project, as already published by [9]. Briefly, a standardized protocol was used to measure the participants’ height and weight. Using a Seca stadiometer (Seca; Hamburg, Germany), the participant’s height was corrected to the nearest 0.1 cm by standing upright on a flat surface without shoes and pressing the back of the heels and the occiput against the stadiometer [31,32]. Furthermore, using a portable Seca scale, participants’ body weight was measured to the nearest 0.1 kg (Seca 770; Hamburg, Germany). Both study sites used the same model of standard calibrated balance. Participants’ BMI was calculated by multiplying their weight in kilograms (kg) by their height in meters squared (m^2^).

#### 2.2.2. Dietary Intake Assessment

Information on the dietary macronutrient status of participants (e-waste recyclers and comparison group) was obtained from the database of the larger project (GEOHealth II project). Each participant’s daily dietary macronutrient intake was documented using a semi-structured 48-h dietary recall guide. This recall was conducted twice (a 24-h recall on each day) to assess the individual’s day-to-day variability due to the variety of foods consumed on different days. To ensure consistency in the survey method across research sites and to further eliminate methodological biases between sites, the GEOHealth II project recruited trained dieticians to collect dietary macronutrient intake data.

#### 2.2.3. Biological Sample Analysis

This study used de-identified archived whole blood samples from the parent project (GEOHealth II project). These blood samples were obtained in clean, enclosed portable stations set up near each study site by a qualified phlebotomist [13]. Approximately 10 mL of blood was collected and placed on a blood tube roller (Micro-Teknik) for 5 min in a trace metal-free BD Vacutainer tube with K₂EDTA. The blood samples were transferred to the laboratory on dry ice and then stored in a −80 °C freezer until analysis.

#### 2.2.4. Blood Glucose (HbA1c) Analysis

HbA1c levels were analyzed in the de-identified whole blood samples using Agilent 1100 High-Performance Liquid Chromatography (HPLC) (Santa Clara, CA, USA), composed of a quaternary pump, auto sampler, diode array detector (DAD), and HP ChemStation Software, CHEM 32). Briefly, a 35 × 4.6 mm PolyCAT A^®^ (3 µm, 1500 Å) column was equilibrated and separation of Hb fractions was achieved by the gradient of mobile phase A (0.02 mol/L Bis-Tris, 2 mmol/L KCN, pH of 6.8) and mobile phase B (0.02 mol/L Bis-Tris, 2 mmol/L KCN, 0.2 mol/L NaCl, pH of 6.6) at a flow rate of 1.5 mL/mL. The DAD detector was used to quantitate eluting peaks at 415 nm.

Four microliters (4 µL) of whole blood sample were aliquoted into 1ml of mobile phase A and gently inverted 8–9 times. Twenty microliters (20 µL) of the hemolyzate were injected. HbA1c levels were identified and quantified based on their retention time relative to the observed retention time for FASC, Control I and Control II obtained from the PolyLC^®^ group. The mean recovery (precision) was 93%, which was well within quality-control protocol target limits (80–120). The normal reference range for HbA1c levels measured after calibration was between 4.5% and 6.9% [33]. The levels of HbA1c measured reflect the mean glucose levels of the e-waste recyclers and the reference population in this study.

### 2.3. Analytical Procedures

#### 2.3.1. Macronutrient Analysis

Using Ghanaian food composition tables, the dietary macronutrient intake data was converted to grams [34,35]. The ESHA F Pro^®^ software (version 7.6) was then used for a comprehensive nutritional analysis to estimate individual macronutrient consumption. The amount of calories, proteins, carbohydrates, saturated fat, monounsaturated fat, polyunsaturated fat, OMEGA 3, OMEGA 6, dietary fiber, cholesterol, and total fat consumed by each participant was obtained from the ESHA F Pro^®^ following nutritional analysis. Findings were then compared to an adult male’s dietary reference intake (DRI) [36].

#### 2.3.2. Laboratory Metal Analysis and Quality Control

1. Metal Analysis

We used our already analyzed baseline levels of Cd and Pb in whole blood of e-waste recyclers and the comparison population using the inductively coupled plasma mass spectrometer (ICPMS; Varian 820MS) [13].

2. Quality Control Measures

Prior to use, all tubes and pipette tips were acid-washed (cleaned, immersed 24 h in 10% hydrochloric acid, then rinsed three times in Milli-Q water). The standard reference materials (SRMs) were obtained from the Institute National de Sante Publique du Quebec. Although analytical standards were employed for each element to develop standard curves and gauge recovery, these reference materials did not cover all the elements we investigated. To calculate analytical precision, each batch run includes duplicate processing (i.e., digestion and ICPMS analysis) of every 10th sample. Finally, procedural blanks were included in each batch run, and the theoretical detection limit was calculated as three times the standard deviation of the mean blank value for each element examined.

### 2.4. Data Processing and Analysis

The outcome variable of interest was blood glucose (HbA1c) levels of the e-waste recyclers and comparison population. However, the exposure variables included blood metals (Pb and Cd) and dietary macronutrient (total calories, carbohydrates, protein, total fat, saturate fat, polyunsaturated fat, monounsaturated fat, OMEGA 3, OMEGA 6, dietary fiber and cholesterol) intake. Levels of Cd, Pb, and HbA1c as well as the amounts of macronutrients consumed the participants were statistically analyzed using descriptive statistics such as means, standard deviation, median and interquartile range. The prevalence of diabetes among e-waste recyclers and the comparison population was reported using descriptive statistics such as proportions and 95% confidence intervals (CIs). The relationship between metal exposures and HbA1c levels were assessed using a simple linear regression model. Similarly, the model was used to investigate the relationship between dietary macronutrient intake and mean HbA1c levels of participants. Next, the joint relationship between metal exposures, dietary macronutrient intake, and blood glucose levels was analyzed using the linear regression model.

## 3. Results

### 3.1. Social-Demographic Characteristics of Study Population

Briefly, the e-waste recyclers were significantly younger (mean: 27.6 ± 0.4 years) and less educated than the comparison population (mean: 30.97 ± 9.92 years) as published by Takyi et al. [13]. While a quarter of the e-waste recyclers had no formal education, more than half of the reference population had completed senior secondary or higher education [13]. More than 50% of the recyclers indicated they worked for about 9 h each day and had been in the industry for about 10 years, with over half of the e-waste recyclers earning 20 to 100 Ghana cedis, while 24% earning less than 20 Ghana cedis [9,10].

### 3.2. Metals Exposure

The mean blood Pb (92.35 ± 63.69 µg/L) levels significantly exceeded the U.S. CDC reference level in about 84% of the e-waste recyclers, compared to the comparison group (40.67 ± 19.12 µg/L) [13]. Conversely, the average Cd levels in blood were significantly higher among the comparison group (0.93 ± 0.64 µg/L) than the recycler group (0.73 ± 0.55 µg/L) [13].

### 3.3. Dietary Macronutrients Intake

Dietary consumption of Omega 6 and Polyunsaturated fatty acids were significantly higher among the comparison group, than the recyclers (Table 1). Although not statistically significant, the comparison group consumed more calories, total fats, and saturated fat [*p* > 0.05]. Similarly, no relationship was identified between dietary macronutrient intake and demographic factors, including daily income accrued, age, religion, job-task performed, as well as educational background. Below is a tabulated detail of the comparison of macronutrient intake between the e-waste recyclers and the comparison group (Table 1).

### 3.4. Prevalence of Diabetes

This study further investigated the prevalence of diabetes among the e-waste recyclers and comparison group (Table 2). Diabetes prevalence was significantly higher among the comparison population (Prev. = 41%, 95% C1: 28.41, 55.26%) compared to the recyclers (Prev. = 31.00%, 95% C1: 22.63, 40.84%) with a (*p* < 0.05) (Table 2). When blood glucose (HbA1c) levels were further categorized into participants with either regulated or unregulated blood glucose levels, 78% (95% CI: 64.83, 87.77) of the comparative population’s blood glucose levels was unregulated (Table 2).

### 3.5. Relationship between Metal Exposures and Blood Glucose Levels

No statistically significant relationship was found between blood Pb or Cd exposure and blood glucose levels of the e-waste recyclers and the comparison group, before and after adjusting for cigarette smoking, alcohol intake, daily income accrued, age, body mass index (BMI), recycler specific job task performed, marital status, and biomass exposure (Appendix A). Similar results were obtained when analysis was limited to the recyclers or the comparison group.

### 3.6. Association between Dietary Macronutrient Intake and Blood Glucose Levels

The consumption of macronutrients like OMEGA 3 and cholesterol from food was associated with significant increases in blood glucose levels of e-waste recyclers and the comparison group (Table 3). Likewise, when the analyses were limited to the recyclers, intake of saturated fat, OMEGA 3, and cholesterol were associated with significant increases in blood glucose levels (*p* < 0.05) (Appendix B). On the contrary, no significant relationships were observed among the comparison population (Appendix C).

### 3.7. Relationship between Metal Exposures, Dietary Macronutrient Intake and Mean Blood Glucose Levels of E-Waste Recyclers and Comparison Group

In the joint model, exposure to Pb (β = 0.009; 95% C: 0.001, 0.017; *p* = *0.03*) coupled with dietary intake of cholesterol (β = 0.009; 95% CI: 0.001, 0.017; *p* = *0.02*) were found to be associated with significant increases in blood glucose levels among the e-waste recyclers and comparison group (Table 4 and Table 5). Similar results were found when the analysis was limited to only recyclers (Appendix D). In a joint model limited to only recyclers, while every 1 mg of cholesterol consumed from food was associated with a 0.7% increase in blood glucose levels (95% CI: 0.001, 0.012; *p* = 0.015; Appendix D), 1 µg/L of Pb was found to significantly augment blood glucose levels by 0.9% (95% CI: 0.001, 0.017; *p* = 0.025; Appendix D). Further, in the model, high Pb exposure and saturated fat intake was associated with significant increases in blood glucose levels in both groups, as well as when limited to recyclers (Appendix D). Dietary intake of OMEGA 3 was also found to be associated with significant increases in blood glucose levels (β = 6.797; 95% CI: 2.960, 10.634; *p* = 0.001) in both groups after Cd exposure (Table 5). Similar results were found when analysis was restricted to the recyclers (Appendix F).

## 4. Discussion

Diabetes is progressively becoming one of the world’s most common non-communicable diseases. Importantly, diabetes remains public health priority, considering its growing global burden and its implications on the quality of life as well as on the economy. Given diabetes is now considered as a rising global health issue, understanding the role of environmental and occupational exposures in the development or progression of diabetes remains critical.

### 4.1. Dietary Macronutrient Intake among E-Waste Recyclers and Comparison Population

Macronutrients are required in prescribed quantity to maintain a healthy body, by preventing illnesses, thus allowing the normal function of the body. The dietary macronutrient consumption data were obtained from the GEOHealth II Study.

To the best of our knowledge, this study is one of the first to investigate the caloric and macronutrient intake of groups exposed environmental toxins such as heavy metals. Given that the e-waste recyclers performed rigorous tasks, it is expected that they would have high-energy demand and will operate high metabolic rates as well. The current findings revealed that both e-waste recyclers and the comparison group did have the appropriate energy balance, given the average caloric amounts consumed. While literature on nutritional intake among groups exposed to toxicants remains limited, previous studies have also documented poor caloric/energy intake among active males. For instance, like the recyclers, while average caloric intake was reported in the RODAMs study [37], both groups did not meet the DRI of 2500–3000 Kcal as set by the American Institute of Medicine [38].

Foods such as whole grain cereals, green leafy vegetables, lean meat, and nuts are food sources rich in OMEGA 6 and polyunsaturated fats. The mean contributions of total fats (including saturated fats, polyunsaturated fats, omega 3, omega 6), carbohydrates, and fiber for both e-waste recyclers and the comparison group were lower than acceptable DRI findings reported by Zhao and Araki et al. [39]. The intake of insufficient quantities of polyunsaturated fats, omega-3, and omega-6 fatty acids could be attributable to increase cardiovascular risk, raised blood glucose levels, which may lead to an onset of diabetes and inflammation [40,41]. However, an adequate dietary intake of fiber could help regulate blood glucose and cholesterol levels in humans, inadequate intake may increase the risk of metabolic diseases including diabetes, hypertension and cancers [42]. Furthermore, the consumption of dietary fiber helps to regulate blood glucose and cholesterol levels in humans [42]. While the recyclers’ protein intake exceeded the DRI, this may be acceptable given their exposure to cuts and muscle loss [43], given the nature of their job [13].

### 4.2. Prevalence of Diabetes among E-Waste Recyclers and Comparison Group

There is evidence of a rising prevalence of diabetes in Ghana. Specifically, studies in the general population in Ghana have reported a diabetes prevalence between 3.3% and 6% among members of the general population, with the incidence increasing with age and being prevalent in urban than rural areas. In this study, diabetes prevalence among the e-waste recyclers (31%) reported here was higher than prevalence reported for residents (22%) of abandoned metal mines in Korea [44]. The variations in documented prevalence could be attributable to occupational differences, metal exposures and sociodemographic variations. In addition, the recycler group was more physically active due to the vigorous job-tasks performed, compared to comparison group, thus were able to maintain blood glucose levels within stipulated reference ranges.

Furthermore, diabetes was more prevalent in the comparison group, which might be linked to a lack of physical activity, resulting from a poorly planned urbanization that lacks a favorable environment for regular exercise, as well as uncontrolled food companies marketing junk food [45]. Some researchers have documented a higher prevalence of diabetes among sedentary workers compared to physically active groups, which is consistent with the current findings, where diabetes prevalence was higher in the comparator population than in the recyclers. For example, Gatimu et al. [44] found that people with a low level of physical activity had a significantly higher prevalence of diabetes (6.74%; 95% CI: 0.42–8.51) than those with a high level of physical activity (2.32%; 95% CI: 0.42–3.15). Aside from physical activity, the higher prevalence of diabetes documented among the comparison group could be linked to the increased environmental emissions, e.g., Cd from emissions of fumes from heavy vehicular traffic, biomass burnings, and dust from unpaved roads [9,10,13].

### 4.3. Relationship between Metal Exposures and Blood Glucose Levels

We found no statistically significant relationship between blood Pb or Cd exposure and blood glucose levels of the e-waste recyclers and the comparison group and this could be linked to the somewhat smaller sample size used in this study. However, several studies have attempted to elucidate the link between heavy metal exposure and diabetes yet reported inconsistent results [46,47,48,49,50]. For instance, after adjusting for age, body mass index, fasting blood glucose, total cholesterol, and triglyceride levels, HbA1c was positively related to the blood Pb levels in a non-diabetic population [51]. Further, Chang et al. [51] also suggested Pb exposure as a risk factor of future development of diabetes. On the contrary, other authors neither observed an overall effect of Pb and Cd plasma levels on HbA1c, nor the interaction effect of the metals on HbA1c [52]. These variations in associations could be attributed to the level of exposure and concentration of metals that humans are exposed to.

Cd levels were higher in comparison group, which is consistent with higher prevalence of diabetes. In other baseline and even large-scale cohort studies [16,47,51], blood Cd was not associated with HbA1c levels. Nonetheless, other authors reported significant associations between Cd exposure and HbA1c levels in humans. For example, Borné et al. [48] cited that the absence of an association between Cd exposure and HbA1c levels could be explained by lower blood Cd levels reported, similar to the levels measured here in this study.

### 4.4. Relationship between Dietary Macronutrient Intake and HbA1c

Increased adherence to traditional dietary sources (meals based on starchy carbohydrates, lower fat and saturated fat content, more fruits and vegetables) has been linked to lower HbA1c levels [53]. This study found positive relationships between total dietary fat and cholesterol intake, consistent with findings reported elsewhere. For example, Churuangsuk et al. [53] reported that higher cholesterol fat and saturated fat intake were linked to higher percent HbA1c among persons aged ≥ 16 years, during eight waves of the UK National Diet and Nutrition Survey (2008–2016) [53]. Likewise, a significant increase in HbA1c, as well as LDL-cholesterol, triglycerides, and C-reactive protein was found to be related to increased fat intake (from 25% to 35%) [54]. Given that high saturated fat diet is linked to insulin resistance and the development of type II diabetes [55], appropriate dietary intake remains necessary to prevent the exacerbation of diabetes and related co-morbidities. Accordingly, consuming more monounsaturated fat and polyunsaturated fat in place of saturated fat or carbohydrate demonstrates a tendency to improve HbA1c levels and may further have positive effects on insulin secretion [6].

OMEGA 3 fatty acids have been shown to have significant impacts on human health in clinical, experimental, and epidemiological studies [56,57]. The low dietary intake of omega-3 fatty acids in e-waste recyclers and the comparison group, might explain the positive relationship between omega-3 fatty acids and HbA1c levels measured in this study. Some studies have shown that omega-3 has potential anti-inflammatory properties and a positive effect of improving insulin sensitivity [58,59,60]. Therefore, people living in both polluted and cleaner cities should be educated on the need for adequate intake of healthy fats like Omega-3 and early supplementation to boost their metabolic health. In addition, some authors have detailed that fish-derived OMEGA 3 fatty acids appear to be particularly protective against pollutant-induced inflammation, and other forms of lipid disposition may serve as a regulatory platform for OMEGA 3 fatty acid-mediated cellular protection [61,62].

Despite gathered evidence that both the quantity and type (quality) of carbohydrate in food affect blood glucose levels, and total amount of carbohydrate consumed serves as primary predictor of glycemic response [63,64], our study found no link between carbohydrate intake and HbA1c. Further, the current study found neither significant associations between carbohydrate intake nor proteins with HbA1c levels.

### 4.5. Relationship between Metal Exposures Dietary Macronutrient Intake and Blood Glucose Levels

Our findings seem to suggest that a poor dietary macronutrient intake combined with metal exposures may increase HbA1c levels in humans. In the joint effect model, for example, high Pb exposure combined with dietary cholesterol and saturated fat consumption was found to be associated with a significant increase in HbA1c levels in both groups as well as when analysis was limited to the recyclers. This could be attributable to poor dietary habits coupled with increased metal exposure associated with informal e-waste recycling.

While Pb is known to trigger oxidative stress by activating reactive oxygen species (ROS), and inhibiting the insulin-signaling pathway, several studies have found that it increased insulin resistance and diabetes [65,66,67]. In humans, macronutrient intake, such as dietary cholesterol and saturated fat, has also been shown to increase insulin resistance [5,68,69]. Although temporality cannot be assumed due to the cross-sectional study design employed, the combined effects of Pb and macronutrients like cholesterol and saturated fats may be linked to increases in participants’ HbA1c levels. Despite the fact that this study measured lower intake of unhealthy fats, such as dietary cholesterol and saturated fats, blood glucose levels were found to be significantly associated with increased Pb exposure. These findings highlight the importance of consistent and appropriate dietary education in both toxicant-exposed groups and general populations to prevent unbalanced dietary fat intake and increased diabetes risks.

Evidence from this study may serve as a basis to help inform dietary guidelines for macronutrients that can improve metabolic health in both exposed groups like recyclers as well as in the general population. To improve the lipid profile and diabetes management, several nutrition-related institutions recommend replacing saturated fat with monounsaturated or polyunsaturated fat [70,71]. Particularly, plant-based meals are not only lower in saturated fats, but also lower in calories and higher in minerals and fiber, a requisite to HbA1c levels in humans.

This study suffered few limitations. We were unable to assume a causal relationship between metal exposure, dietary macronutrient intake, and blood glucose levels, since we used a cross-sectional study design. Secondly, data on physical activity levels of the participants were not captured, although it has been shown to influence both nutritional and metabolic status. Nevertheless, we provide useful firsthand data on the prevalence of diabetes among toxicant-exposed groups in Ghana, such as e-waste recyclers. Further, and to the best of our knowledge, this study is the first to investigate the joint relationship between metal exposures, dietary macronutrient intake, and blood glucose levels among male e-waste recyclers in Ghana and elsewhere.

## 5. Conclusions

In addition to the adequate intake of proteins, this study highlights the inadequate intake of macronutrients, e.g., carbohydrates, fiber, and polyunsaturated fats (OMEGA 3 and OMEGA 6), among e-waste recyclers and the comparison group. The prevalence of diabetes was higher among the comparison group, given their sedentary job nature, relative to recyclers who performed vigorous work-tasks. Although unhealthy fats, such as dietary cholesterol and saturated fats, were inadequately consumed, the exposure to Pb and Cd, together with a reduced intake of these fats, may still increase the risk of diabetes among both e-waste recyclers and the general population. It is suggested that mechanistic studies be designed and implemented to focus on how macronutrients and pollutants interfere with glucose metabolism. Our findings suggest the continuous need for the intensification of public health education and promotion focusing on adequate nutrient intake and healthy eating strategies among both e-waste recyclers and general populations in Ghana and elsewhere.

## Figures and Tables

**Table 1:** Daily Dietary macronutrient intake of e-waste recyclers and comparison group.

	E-waste Recycler		Comparison Group		*p*-Value
	Mean ± SD	Median (IQR)	Mean ± SD	Median (IQR)	
Total Calories (kcal)	2050.05 ± 673.05	1996.75 ± 28.89	2067.34 ± 793.50	1976.38 ± 1165.5	0.75
Carbohydrates (g)	305.28 ± 105.93	295 ± 124.5	289.901 ± 118.74	271 ± 175	0.39
Proteins (g)	72.15 ± 29.89	67.98 ± 33.83	65.30 ± 27.20	59.63 ± 37.7	0.10
Total Fats (g)	66.57 ± 32.46	61.48 ± 32.93	74.32 ± 50.63	62.9 ± 37.85	0.64
Saturated Fats (g)	6.25 ± 4.52	5.45 ± 5.22	6.58 ± 4.80	5.96 ± 6.34	0.73
Mono Fats (g)	6.99 ± 6.13	5.62 ± 4.92	9.04 ± 8.33	7.44 ± 7.26	0.10
Poly Fats (g)	3.40 ± 5.00	1.94 ± 2.37	4.55 ± 5.02	2.95 ± 3.77	0.02 *
Omega 3 (g)	0.16 ± 0.14	0.12 ± 0.18	0.19 ± 0.17	0.14 ± 0.19	0.46
Omega 6 (g)	1.89 ± 2.50	1.21 ± 1.66	2.92 ± 3.86	1.88 ± 1.95	0.02 *
Cholesterol (mg)	123.12 ± 97.21	91.5 ± 132.15	99.75 ± 77.56	91 ± 91.4	0.28
Dietary Fibre (g)	19.32 ± 11.11	17.58 ± 13.5	20.89 ± 13.97	17.43 ± 14.98	0.96

*p*-Value notation: *p* < 0.05 * for which reason their values are highlighted in bold.

**Table 2:** Prevalence of diabetes among e-waste recyclers and comparison group.

Blood Glucose Categories	E-Waste Recyclers % [95% CI]	Comparison Group % [95% CI]	χ^2^ (*p*-Value)
**Blood Glucose Category 1**			6.20 (0.05)
Low blood glucose levels (<4.5%)	27.00 [19.10, 36.69]	37.26 [24.81, 51.65]	
Normal Blood glucose levels (4.5–6.9%)	42.00 [32.63, 51.99]	21.57 [12.23, 35.17]	
High blood glucose levels (>6.9%)	31.00 [22.63, 40.84]	41.18 [28.41, 55.26]	
**Blood Glucose Category 2**			**6.19 (0.01)**
Regulated blood glucose (4.5–6.9%)	42.00 [32.63, 51.99]	21.57 [12.23, 35.17]	
Unregulated blood glucose levels (<4.5 and >6.9%)	58.00 [48.01, 67.37]	78.43 [64.83, 87.77]	

The blood glucose categories are defined by the American Diabetes Association [33]. *p*-Value notation: *p* < 0.05 for which reason their values are highlighted in bold.

**Table 3:** Association between dietary macronutrient intake and mean blood glucose levels among e-waste recyclers and comparison group.

Dietary Macronutrients	Mean Blood Glucose Levels (HbA1c (%)) β [95% CI]
Total Calories (kcal)	−0.0001 [−0.001, 0.0004]
Carbohydrates (g)	−0.0004 [−0.004, 0.003]
Proteins (g)	0.001 [−0.013, 0.014]
Total Fats (g)	−0.003 [−0.012, 0.006]
Saturated Fats (g)	0.061 [−0.025, 0.147]
Mono Fats (g)	0.005 [−0.048, 0.058]
Poly fats (g)	0.015 [−0.054, 0.085]
Omega 3 (g)	3.397 * [1.010, 5.784]
Omega 6 (g)	0.089 [−0.030, 0.209]
Cholesterol (mg)	0.005 * [.001, 0.009]
Dietary Fibre (g)	−0.013 [−0.042, 0.018]

*p*-value notation: *p* < 0.05 * for which reason their values are highlighted in bold.

**Table 4:** Relationship between Pb exposures, macronutrient intake and mean blood glucose levels of e-waste recyclers and comparison group.

Variables	Mean Blood Glucose Levels (HbA1c (%)) β (95% CI)
B-Pb	0.007 [−0.002, 0.015]
Total Calories	−0.001 [−0.002, 0.001]
B-Pb	0.007 [−0.001, 0.015]
Carbohydrate (g)	−0.005 [−0.012, 0.001]
B-Pb	0.007 [−0.001, 0.015]
Protein (g)	0.0001 [−0.030, 0.030]
B-Pb	0.007 [−0.001, 0.015]
Total Fats (g)	−0.002 [−0.021, 0.016]
B-Pb	0.008 * [0.00004, 0.016]
Saturated Fats (g)	0.136 * [0.015, 0.258]
B-Pb	0.008 [−0.0005, 0.016]
Mono Fats (g)	0.044 [−0.037, 0.125]
B-Pb	0.008 [−0.001, 0.016]
Poly fats (g)	0.037 [−0.058, 0.132]
B-Pb	0.007 * [0.0002, 0.015]
Omega 3 (g)	6.88 * [3.247, 10.523]
B-Pb	0.008 [−0.0001, 0.016]
Omega 6 (g)	0.152 [−0.035, 0.339]
B-Pb	0.009 * [0.001, 0.017]
Cholesterol (mg)	0.007 * [0.001, 0.012]
B-Pb	0.007 [−0.001, 0.015]
Dietary Fibre (g)	−0.016 [−0.070, 0.037]

*p*-Value notation: *p* < 0.05 * for which reason their values are highlighted in bold. Abbreviations: B-Pb: Blood Lead; Random effects adjustments were made for cigarette smoking, alcohol intake, daily income accrued, age, BMI, weight, educational status, years of work, marital status and mean probability of adequacy.

**Table 5:** Relationship between Cd exposure, macronutrient intake and blood glucose levels of e-waste recyclers and comparison group.

Variables	Blood Glucose Levels (HbA1c (%)) β (95% CI)
B-Cd	0.250 [−0.879, 1.379]
Total Calories	−0.001 [−0.002, 0.0004]
B-Cd	0.223 [−0.900, 1.346]
Carbohydrate (g)	−0.005 [−0.012, 0.002]
B-Cd	0.242 [−0.903, 1.388]
Protein (g)	−0.001 [−0.032, 0.029]
B-Cd	0.273 [−0.877, 1.423]
Total Fats (g)	−0.004 [−0.023, 0.015]
B-Cd	0.175 [−0.933, 1.284]
Saturated Fats (g)	1.121 [−0.005, 0.246]
B-Cd	0.254 [−0.885, 1.394]
Mono Fats (g)	0.029 [−0.053, 0.111]
B-Cd	0.251 [−0.892, 1.395]
Poly fats (g)	0.018 [−0.077, 0.113]
B-Cd	−0.088 [−1.133, 0.958]
Omega 3 (g)	6.797 * [2.960, 10.634]
B-Cd	0.232 [−0.895, 1.359]
Omega 6 (g)	0.121 [−0.069, 0.312]
B-Cd	0.265 [−0.840, 1.370]
Cholesterol (mg)	0.005 [−0.0001, 0.011]
B-Cd	0.335 [−0.075, 1.483]
Dietary Fibre (g)	−0.020 [−0.075, 0.035]

*p*-Value notation: *p* < 0.05 * for which reason their values were boldened. Abbreviations: B-Cd: Blood Cadmium; Random effects adjustments were made for cigarette smoking, alcohol intake, daily income accrued, age, BMI, weight, educational status, years of work, marital status and mean probability of adequacy.

## Data Availability

The data presented in this study are available in our already published papers including Takyi et al. [13], and Takyi et al. [28].

## Appendix A

**Table A1:** Relationship between metals exposures and blood glucose levels of e-waste recyclers and comparison group.

Metals (µg/L)	Blood Glucose Levels (HbA1c (%)) β [95% CI]
*E-waste recyclers and Comparison Group*	
B-Pb	0.003 [−0.003, 0.009]
B-Cd	−0.193 [−0.854, 0.467]
*E-waste recyclers only*	
B-Pb	0.002 [−0.004, 0.008]
B-Cd	0.109 [−0.859, 1.077]
*Comparison group only*	
B-Pb	0.028 [−0.010, 0.066]
B-Cd	−0.589 [−1.740, 0.564]

*p*-value notation: *p* < 0.05, Abbreviations: B-Pb: Blood Lead; B-Cd: Blood Cadmium; Random effects adjustments were made for cigarette smoking, alcohol intake, daily income accrued, age, BMI, recycler specific job task performed, marital status, & biomass exposure.

## Appendix B

**Table A2:** Relationship between dietary macronutrient intake and blood glucose levels of e-waste recyclers only.

Dietary Macronutrients	Blood Glucose Levels (HbA1c (%)) β (95% CI)
Total Calories (g)	−0.0004 [−0.001, 0.0003]
Carbohydrates (g)	−0.002 [−0.007, 0.002]
Proteins (g)	−0.008 [−0.024, 0.008]
Total Fats (g)	−0.002 [−0.016, 0.010]
Saturated Fats (g)	**0.097 *** [0.0002, 0.193]
Mono Fats (g)	0.020 [−0.046, 0.087]
Poly fats (g)	0.019 [−0.061, 0.099]
Omega 3 (g)	**4.800 *** [1.852, 7.748]
Omega 6 (g)	0.083 [−0.076, 0.243]
Cholesterol (mg)	**0.004 *** [0.0003, 0.009]
Dietary Fibre (g)	−0.003 [−0.042, 0.036]

*p*-value notation: *p* < 0.05 * for which reason their values were boldened.

## Appendix C

**Table A3:** Relationship between macronutrient intake and blood glucose levels of comparison group.

Dietary Macronutrients	Blood Glucose Levels (HbA1c (%)) β (95% CI)
Total Calories (g)	0.000 [−0.001, 0.001]
Carbohydrates (g)	0.003 [−0.004, 0.010]
Proteins (g)	0.019 [−0.009, 0.047]
Total Fats (g)	−0.001 [−0.016, 0.015]
Saturated Fats(g)	−0.043 [−0.242, 0.157]
Mono Fats (g)	−0.019 [−0.123, 0.086]
Poly fats (g)	0.019 [−0.147, 0.185]
Omega 3 (g)	1.527 [−3.214, 6.268]
Omega 6 (g)	0.073 [−0.161, 0.306]
Cholesterol (mg)	0.008 [−0.002, 0.019]
Dietary Fibre (g)	−0.022 [−0.078, 0.034]

*p*-Value notation: *p* < 0.05.

## Appendix D

**Table A4:** Relationship between Pb exposure, macronutrient intake and blood glucose levels of e-waste recyclers.

Variables	Blood Glucose Levels (HbA1c (%)) β (95% CI)
B-Pb	0.007 [−0.002, 0.015]
Total Calories	−0.001 [−0.002, 0.001]
B-Pb	0.007 [−0.001, 0.015]
Carbohydrate (g)	−0.005 [−0.012, 0.001]
B-Pb	0.007 [−0.001, 0.015]
Protein (g)	−0.0001 [−0.030, 0.030]
B-Pb	0.007 [−0.001, 0.015]
Total Fats (g)	−0.002 [−0.021, 0.016]
B-Pb	**0.008 *** [0.00004, 0.016]
Saturated Fats (g)	**0.136 *** [0.015, 0.258]
B-Pb	0.008 [−0.0005, 0.016]
Mono Fats (g)	0.044 [−0.037, 0.125]
B-Pb	0.008 [−0.001, 0.009]
Poly fats (g)	0.037 [−0.058, 0.132]
B-Pb	**0.007 *** [0.0002, 0.015]
Omega 3 (g)	**6.884 *** [3.247, 10.523]
B-Pb	0.008 [−0.0001, 0.016]
Omega 6 (g)	0.152 [−0.035, 0.339]
B-Pb	**0.009 *** [0.001, 0.017]
Cholesterol (mg)	**0.007 *** [0.001, 0.012]
B-Pb	0.007 [−0.001, 0.015]
Dietary Fibre (g)	−0.016 [−0.070, 0.037]

*p*-Value notation: *p* < 0.05 * for which reason their values were boldened; Abbreviations: B-Pb: Blood Lead Random effects adjustments were made for cigarette smoking, alcohol intake, daily income accrued, age, BMI, weight, educational status, years of work, marital status and mean probability of adequacy.

## Appendix E

**Table A5:** Relationship between Cd exposure, macronutrient intake and blood glucose levels of e-waste recyclers.

Variables	Blood Glucose Levels (HbA1c (%)) β (95% CI)
B-Cd	0.250 [−0.879, 1.379]
Total Calories	−0.001 [−0.002, 0.0004]
B-Cd	0.223 [−0.900, 1.346]
Carbohydrate (g)	−0.005 [−0.012, 0.002]
B-Cd	0.242 [−0.903, 1.388]
Protein (g)	−0.001 [−0.032, 0.029]
B-Cd	0.273 [−0.877, 1.423]
Total Fats (g)	−0.004 [−0.023, 0.015]
B-Cd	0.175 [−0.933, 1.284]
Saturated Fats (g)	1.121 [−0.005, 0.246]
B-Cd	0.254 [−0.885, 1.394]
Mono Fats (g)	0.029 [−0.053, 0.111]
B-Cd	0.251 [−0.892, 1.395]
Poly fats (g)	0.018 [−0.077, 0.113]
B-Cd	−0.088 [−1.133, 0.958]
Omega 3 (g)	**6.797 *** [2.960, 10.634]
B-Cd	0.232 [−0.895, 1.359]
Omega 6 (g)	0.121 [−0.069, 0.312]
B-Cd	0.265 [−0.840, 1.370]
Cholesterol (mg)	0.005 [−0.0001, 0.011]
B-Cd	0.335 [−0.814, 1.483]
Dietary Fibre (g)	−0.020 [−0.075, 0.035]

*p*-Value notation: *p* < 0.05 * for which reason their values were boldened; Abbreviations: B-Cd: Blood Cadmium; Random effects adjustments were made for cigarette smoking, alcohol intake, daily income accrued, age, BMI, weight, educational status, years of work, marital status and mean probability of adequacy.

## Appendix F

**Table A6:** Relationship between Pb exposure, macronutrient intake and blood glucose levels of comparison group.

Variables	Blood Glucose Levels (HbA1c (%)) β (95% CI)
B-Pb	0.026 [−0.013, 0.065]
Total Calories	0.0002 [−0.001, 0.001]
B-Pb	0.025 [−0.014, 0.065]
Carbohydrate (g)	0.001 [−0.006, 0.008]
B-Pb	0.002 [−0.004, 0.008]
Protein (g)	−0.024 [−0.014, 0.062]
B-Pb	0.028 [−0.011, 0.066]
Total Fats (g)	−0.001 [−0.016, 0.015]
B-Pb	[0.027 [−0.011, 0.066]
Saturated Fats (g)	−0.032 [−0.229, 0.165]
B-Pb	0.027 [−0.011, 0.065]
Mono Fats (g)	−0.011 [−0.114, 0.093]
B-Pb	0.028 [−0.010, 0.067]
Poly fats (g)	0.024 [−0.140, 0.187]
B-Pb	0.030 [−0.010, 0.070]
Omega 3 (g)	1.329 [−3.470, 6.134]
B-Pb	0.029 [−0.011, 0.068]
Omega 6 (g)	0.0536 [−0.179, 0.286]
B-Pb	0.033 [−0.004, 0.070]
Cholesterol (mg)	0.010 [−0.001, 0.020]
B-Pb	0.029 [−0.010, 0.067]
Dietary Fibre (g)	−0.019 [−0.075, 0.037]

*p*-Value notation: *p* < 0.05; Abbreviations: B-Pb: Blood Lead; B-Cd: Blood Cadmium; Random effects adjustments were made for cigarette smoking, alcohol intake, daily income accrued, age, BMI, recycler specific job task performed, marital status.

## Appendix G

**Table A7:** Relationship between Cd exposure, macronutrient intake and blood glucose levels of comparison group.

Variables	Blood Glucose Levels (HbA1c (%)) β (95% CI)
B-Cd	−0.541 [−1.720, 0.638]
Total Calories	0.0003 [−0.001, 0.001]
B-Cd	−0.558 [−1.726, 0.609]
Carbohydrate (g)	0.002 [−0.005, 0.009]
B-Cd	−0.456 [−1.623, 0.711]
Protein (g)	0.017 [−0.012, 0.046]
B-Cd	−0.599 [−1.778, 0.580]
Total Fats (g)	−0.001 [−0.017, 0.014]
B-Cd	−0.580 [−1.750, 0.589]
Saturated Fats (g)	−0.039 [−0.239, 0.160]
B-Cd	−0.585 [−1.755, 0.586]
Mono Fats (g)	−0.018 [−0.122, 0.086]
B-Cd	−0.624 [−1.812, 0.564]
Poly fats (g)	0.028 [−0.140, 0.197]
B-Cd	−0.525 [−1.728, 0.679]
Omega 3 (g)	0.674 [−4.221, 5.568]
B-Cd	−0.543 [−1.740, 0.654]
Omega 6 (g)	0.048 [−0.189, 0.286]
B-Cd	−0.490 [−1.632, 0.653]
Cholesterol (mg)	0.008 [−0.003, 0.018]
B-Cd	−0.621 [−1.789, 0.548]
Dietary Fibre (g)	−0.019 [−0.076, 0.039]

*p*-Value notation: *p* < 0.05, Abbreviations: B-Pb: Blood Lead; B-Cd: Blood Cadmium; Random effects adjustments were made for cigarette smoking, alcohol intake, daily income accrued, age, BMI, recycler specific job task performed, marital status.
